# Identification of Risk Factors for Suicidal Ideation and Attempt Based on Machine Learning Algorithms: A Longitudinal Survey in Korea (2007–2019)

**DOI:** 10.3390/ijerph182312772

**Published:** 2021-12-03

**Authors:** Junggu Choi, Seoyoung Cho, Inhwan Ko, Sanghoon Han

**Affiliations:** 1 Yonsei Graduate Program in Cognitive Science, Yonsei University, Seoul 03722, Korea; junggu.choi@yonsei.ac.kr (J.C.); seoyoung.cho@yonsei.ac.kr (S.C.); 2 Department of Psychology, Yonsei University, Seoul 03722, Korea; inhwan.ko@yonsei.ac.kr

**Keywords:** suicidal risk factor, suicide attempt, suicidal ideation, machine learning algorithm, longitudinal survey dataset

## Abstract

Investigating suicide risk factors is critical for socioeconomic and public health, and many researchers have tried to identify factors associated with suicide. In this study, the risk factors for suicidal ideation were compared, and the contributions of different factors to suicidal ideation and attempt were investigated. To reflect the diverse characteristics of the population, the large-scale and longitudinal dataset used in this study included both socioeconomic and clinical variables collected from the Korean public. Three machine learning algorithms (XGBoost classifier, support vector classifier, and logistic regression) were used to detect the risk factors for both suicidal ideation and attempt. The importance of the variables was determined using the model with the best classification performance. In addition, a novel risk-factor score, calculated from the rank and importance scores of each variable, was proposed. Socioeconomic and sociodemographic factors showed a high correlation with risks for both ideation and attempt. Mental health variables ranked higher than other factors in suicidal attempts, posing a relatively higher suicide risk than ideation. These trends were further validated using the conditions from the integrated and yearly dataset. This study provides novel insights into suicidal risk factors for suicidal ideations and attempts.

## 1. Introduction

In the past 10 years, approximately 800,000 people committed suicide annually [1,2,3]. Suicidal mortality is considered a critical factor for both social and public health [4,5,6]. Many previous studies have suggested that death by suicide has socioeconomic and psychological consequences, burdening members of society [7,8,9]. Paul et al. [10] analyzed the social and economic burden of suicide in the Hong Kong SAR. In addition, Shumona et al. [11] attempted to validate suicidal risk factors and their impact in rural Bangladesh. They suggested that the burden of suicide is a major health problem. In the current COVID-19 pandemic, depression and suicide are important mental health care challenges [12,13,14].

To solve problems related to suicide, researchers have attempted to identify its underlying factors. Using systematic reviews, Elizabeth et al. [15] proposed several risk factors associated with suicidal self-directed violence among veterans living in the US. In addition, Mościcki [16] investigated the contributions of sociodemographic factors (e.g., age, gender, race, and socioeconomic status) to suicidal risk based on epidemiologic studies. Madelyn et al. [17] demonstrated the risk of psychosocial factors associated with suicide in children and adolescents. In their final analysis, socioenvironmental circumstances were confirmed to be a significant factor in teenage suicide risk.

Previous studies have focused on two categories of risk factors: “psychiatric or clinical” and “economic or social elements”, which were determined to be influential factors in related studies. However, the focus on these variables in related studies differed depending on the topic of interest or the population studied. First, when conducting research with psychiatric patient groups, major associations between the clinical variables and suicidal risk were found. For example, Gregory et al. [18] attempted to determine the risk factors for psychiatric outpatient groups. Various variables, including the current intensity of the patients’ specific attitudes and behaviors, were collected from a total of 6981 patients. The contributions of psychiatric variables to the risk of eventual suicide were identified among diverse categories of variables, including clinical and economic factors. Second, when conducting research on patient groups from specific population (e.g., children or adolescents), socioeconomic factors were found to be major influencing factors. Esben et al. [19] estimated the risk factors for young people living in Denmark. Their participants answered survey questions detailing mental illness, employment, and income. Having parents with a low socioeconomic status was found to be a relatively high risk factor for suicide among young people. To reflect the diverse characteristics of the population as much as possible, we analyzed large-scale and longitudinal datasets collected from the Korean public. Moreover, in terms of a multivariate analysis, diverse variables, including both clinical and socioeconomic factors, were used to determine associations between the factors.

In addition, the study groups were divided according to suicide-related events. Nock and Banaji [20] predicted suicidal ideation and suicide attempts based on test results. Their participants were divided into three groups (i.e., non-suicidal, suicide ideators, and recent suicide attempters), and the analysis results were compared. To examine the different characteristics of suicide attempters and suicide completers, Konrad [21] evaluated their medical fitness, personality, and clinical characteristics. Matthew et al. [22] compared the risk factors of suicidal ideation, plans, and attempts through a cross-national analysis. Some researchers analyzed the effects of risk factors by comparing multiple datasets. To investigate the effects of factors contributing to suicide events, namely, suicide ideation, planning, attempts, and completion, datasets of multiple events were compared in numerous previous studies [23,24,25].

To identify risk factors based on participant characteristics, diverse methodologies for analysis, including statistical modeling, have been applied. Gutierrez et al. [26] used structural equation modeling to determine the relationships between candidate risk factors. A chi-square analysis was subsequently used to validate the modeling results. Berman [27] utilized descriptive statistics (e.g., mean and proportions) to summarize patient characteristics. Fisher’s exact test with a two-tailed test was used to compare the clinical characteristics of the patient groups. Using Pearson’s correlation analysis, Park and Jang [28] evaluated the association between suicide rates and risk factors among Korean adolescents.

In recent studies, machine learning algorithms have shown better performance than traditional statistical methods for structural type datasets (e.g., datasets collected from surveys). Subramani et al. [29] attempted to forecast the risk of diabetes through electronic medical records (EMRs) with machine learning classifiers, such as support vector machines and decision trees. Sangita et al. [30] used a logistic regression model to investigate the nutritional status of children using Indian demographic and health survey datasets. From these studies, it was confirmed that machine learning models have sufficient capability to analyze structural datasets.

Machine learning models have been applied in many previous studies to investigate the risk factors for suicide. De la Garza et al. [31] attempted to determine the risk factors for nonfatal suicide attempts. They utilized the importance of features in trained algorithms to determine the emphasized variables. Samah et al. [32] proposed a machine learning-based framework to detect potential suicide risk factors using text datasets collected from Twitter. The decision tree model and K-means clustering algorithms were applied to classify the risk levels. The clustering results and classification performances were used to identify the risk factors for suicide. The authors reported that words related to feelings of depression and self-harm were important in classifying suicide risk levels.

Suicidal ideation and attempt are compared in this study to analyze and determine the relative influence of risk factors. The degree of risk for suicide ideation was set as a low-risk condition, and that for suicide attempt was set as a relatively high-risk condition. The importance of different risk factors for a suicide attempt was examined in the high-suicide-risk group based on the underlying effects (e.g., economic and psychological burden). Machine learning algorithms were used to compare the influence of risk factors for suicidal ideation and suicide attempts separately with that of suicide risk (i.e., suicide ideation was classified as a low suicide risk, and suicide attempt was classified as a relatively high suicide risk). The longitudinal dataset obtained from the general population of Korea was utilized to determine the risk factors. In addition, a new risk-factor score based on feature importance and the rank of the variable was proposed to confirm their importance in suicidal ideation and attempt.

The major contributions of our research are as follows. First, to reflect the various characteristics of the study population, large-scale (*n* = 215,522) and longitudinal (from 1998 to 2019) datasets collected in Korea were used. Second, machine learning algorithms were applied to detect the inherent patterns and factors in the dataset associated with suicide ideation and suicidal attempts from the dataset. Third, a novel risk-factor score was proposed and applied to compare the importance of the factors using the results of the machine learning classifiers. Finally, the risks for suicide ideation and attempt were separately validated based on a comparison between the risks of both suicide ideation and attempt.

## 2. Materials and Methods

### 2.1. Overview

**Figure 1:**
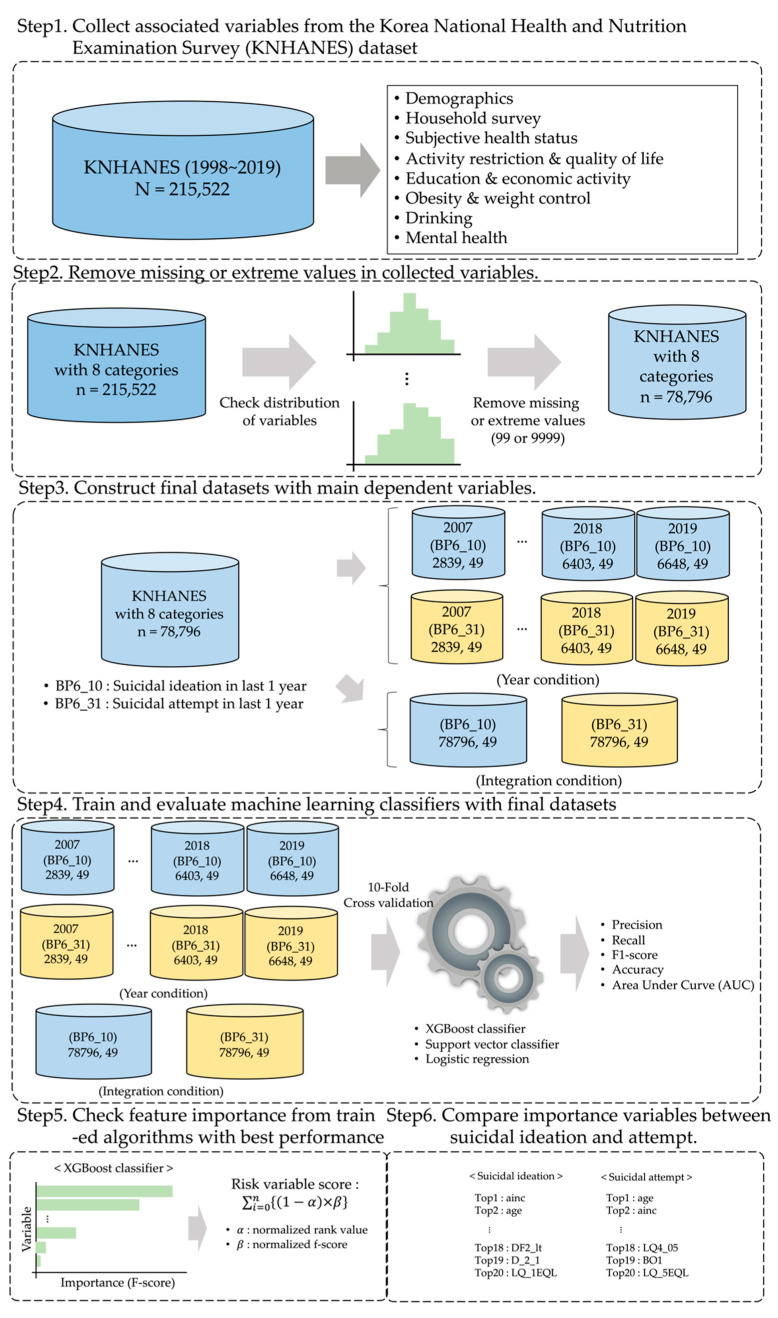
Overview of the research scheme adopted in this study.

To determine the major risk factors for suicidal ideation and attempts, our research was divided into six steps. First, the associated variables were collected from the Korea National Health and Nutrition Examination Survey (KNHANES) dataset. Second, missing or extreme values among the collected variables were removed to better reflect the characteristics of the participants. Third, the final datasets were constructed based on the main dependent variables (i.e., suicide ideation and suicide attempts). Fourth, three machine-learning algorithms were trained and evaluated using previously organized datasets. Fifth, the importance of each feature was determined using the model with the best classification performance. Finally, the score of each variable was calculated and compared to reveal the differences between ideation and suicide attempts. The detailed steps are shown in Figure 1.

### 2.2. Data Source

In this study, the open-source KNHANES dataset released by the Korea Disease Control and Prevention Agency (KDCA) [33] was utilized to compare the risk factors of suicidal ideation and attempts. KNHANES is a longitudinal survey that investigates the health status, health-related awareness and behavior, and nutritional status of people living in Korea. This survey is conducted annually by the KDCA. The first survey was conducted in 1998 and was repeated at three-year intervals until 2005. Since 2007, surveys have been conducted annually. A total of 216,815 people participated in the survey from 1998 to 2019. The original datasets collected from the surveys are available to the public. The KNHANES dataset was constructed using nine categories of survey variables. A detailed list of the categories is presented in Table 1.

The survey results were stored in two datafiles. In the first file, survey variables for the health behavior, blood test, blood pressure test, and hand grip test results were included. The other test results (dietary life, food safety, food intake, and dietary life evaluation) were stored in a second datafile. All variables in the data files can be merged based on the participant ID.

Additionally, the public dataset on suicide rate released by the Korean Statistical Information Service (KOSIS) was utilized to investigate the effects of suicide rate on the associated risk factors. In this study on the KNHANES datasets from 1998 to 2019, twenty-one suicide rate values were used for the analysis.

### 2.3. Data Preprocessing

#### 2.3.1. Collection and Selection of Associated Variables from Datasets

To reflect the various characteristics of the participants, all available datasets in KNHANES (i.e., from 1998 to 2019) were utilized. In addition, the associated variables, including socioeconomic and psychiatric variables, were selected from the datasets. Among the nine categories of variables, the variables for demographics, household, subjective health status, activity restriction and quality of life, education and economic activity, obesity and weight control, drinking, and mental health were extracted to identify major suicidal risk factors. The dimensions of the original dataset were (215,522, 851). After the extraction of relevant variables, the dimensions of the remaining dataset were (78,796, 58). The baseline characteristics of the datasets are listed in Table 2.

#### 2.3.2. Removal of Missing or Extreme Data in the Dataset

**Figure 2:**
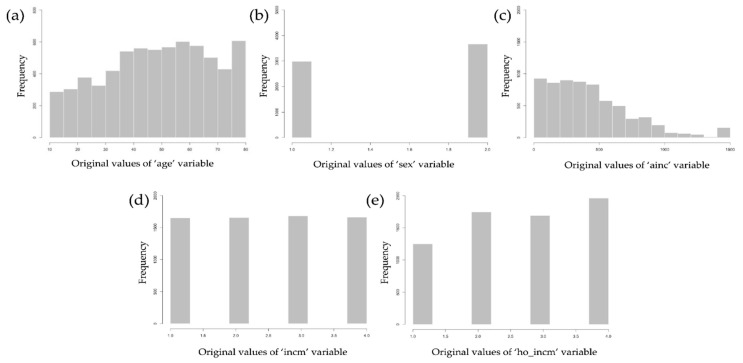
Distributions of variables used in our study: (**a**) distribution of “age”, (**b**) distribution of “sex”, (**c**) distribution of “ainc”, (**d**) distribution of “incm”, and (**e**) distribution of “ho_incm”.

The distributions of the variables were analyzed to remove missing or extreme values from the data. In the KNHANES dataset, the missing values were coded as 99 or 9999. To reflect the exact response to each variable, the distribution of each variable was examined using histograms. After removing the variables in more than half of the responses, 48 variables remained. The distribution of the variables used in our study is shown in Figure 2.

After verifying the distribution of each variable in the datasets, the remaining variables in the datasets from 1998 to 2019 were compared. For the comparison, common variables were selected from a total of 13 datasets for the years 2007 to 2019. Datasets from 1998 to 2006, with uncommon variables, were excluded. The dimensions and number of participants for the 13 datasets from 2007 to 2019 are listed in Table 3.

#### 2.3.3. Generation of the Final Datasets for the Evaluation of Machine Learning Classifiers

To verify the difference between suicidal ideation and attempt, we set “BP6_10” (suicidal ideation within the last year) and “BP6_31” (suicide attempts within the last year) as the dependent variables. Other variables were applied to the machine learning classifiers as independent variables. The dataset was divided by year to compare the analysis results individually, and the integrated dataset was further analyzed, regardless of the year, to investigate differences in the risk factors. The suicide rates per year in the dataset were used to determine the effect of suicide rate on major risk factors for suicide.

Based on the aforementioned conditions used for the comparison in our study, the comparison of the experimental results was performed under a total of 28 conditions (two dependent conditions in 14 datasets from 2007 to 2019). The datasets for the 28 conditions were divided into training and test datasets with an 8:2 ratio.

### 2.4. Training and Evaluation of Machine Learning Classification Algorithm

As described in the previous section, the machine learning classifiers were applied to the 28 conditions in the datasets for a comparison. In this study, the XGBoost classifier, support vector classifier, and logistic regression were used. According to the binary characteristics of the dependent variables (“BP6_10” and “BP6_31”), all algorithms performed binary classification tasks under all experimental conditions. The BP6_10 and BP6_31 variables were collected from different survey questions. The BP6_10 values consisted of binary answers to “Have you thought about suicide within the last year?” The BP6_31 values consisted of binary answers to “Have you attempted suicide within the last year?” Participants answered both questions in binary format (i.e., yes = 1; no = 0). The importance of the features was recorded for the test dataset to identify important features among the independent variables using the trained algorithms.

A random search was conducted to determine the optimal hyperparameters of the three ML classifiers, as listed in Table 4. In addition, to prevent overfitting of the classification algorithms, 10-fold cross-validations were applied when training the algorithms.

### 2.5. Calculation of the Risk-Factor Score from Feature Importance Results

From the evaluation of the trained algorithms on the test datasets, the importance of each feature was determined for the best performing classifier. The importance scores (e.g., F-score for XGBoost classifier, coefficients for SVC, and logistic regression) and rank for each variable were confirmed through the analysis of feature importance. To simultaneously consider both results (importance score and rank of the variable), a new quantified score was devised for the risk of each variable by integrating the importance and rank. The proposed score for each variable was calculated using the following formula:

(1) $$\mathrm{Risk}\text{-}\mathrm{factor}\;\mathrm{score}=\frac{\sum_{i=0}^{n}\left(1-\alpha_{i}\right)\times \beta_{i}}{n}$$

where α denotes a normalized rank between 0 and 1, and β represents the normalized importance score. In our study, a 10-fold cross-validation was used for training and evaluating the algorithms. As a result, 10 evaluation sets were used for each experiment, and the same results for the evaluation of the variables were observed. Finally, to create a single score, the values for multiple variables were averaged.

### 2.6. Machine Learning (ML) Classification Algorithms

#### 2.6.1. XGBoost Classifier

The XGBoost classifier is a supervised learning algorithm based on gradient boosting methods [34]. This classification model was constructed using classification and regression tree (CART) methods. In addition, it is an ensemble model composed of several decision-tree models. The objective function of this algorithm with ensemble learning is as follows:

(2) $$\mathrm{Objective}\;\mathrm{function}=\overset{n}{\underset{i=1}{\sum }}L\left(y_{i},y_{i}^{\prime }\right)+\overset{m}{\underset{j=1}{\sum }}\Omega \left(f_{j}\right)$$

(3) $$\mathrm{where}\;y_{i}^{\prime }=\sum_{k=1}^{K}f_{k}\left(x_{i}\right)$$

In the above function, the first formula indicates the objective function of the XGBoost classifier. Function L is the loss function for the algorithm evaluation. In addition, the function Ω denotes the regularization term and mean complexity of the models. $y_{i}^{\prime }$ values are calculated using the kth decision tree, represented by $f_{k}$. In this study, $y_{i}$ set class labels can denote suicidal ideation or attempts (e.g., coded with 0 or 1 for suicidal ideation or attempt, respectively, within the past year).

#### 2.6.2. Support Vector Classifier

The second classification algorithm used in this study is the support vector classifier (SVC) with radial basis function (RBF) kernels [35]. This classification algorithm divides the feature space into hyperplanes that are separated using class labels. A radial basis function kernel with non-linear characteristics was applied to the classifiers. In previous studies, linear kernels were used for binary classification tasks [36]. Here, it was experimentally verified that the SVC model with the RBF kernel resulted in better model performance compared with the model with a linear kernel. In addition, completely participant-separated datasets were used to avoid overfitting the classifiers.

#### 2.6.3. Logistic Regression

The last classification algorithm used in our study was the logistic regression model. This classification model calculates the log-odds value of each variable and applies a sigmoid function to each result [37]. The probability that the data belong to the corresponding class was calculated using this model. A probability higher than 0.5 indicates that the variable was classified as being a risk factor for having had suicidal ideation or attempt in the past year. The basic form of this model including the variables and classes for suicidal ideation or attempts is as follows:

(4) $$F\left(z\right)=E\left(\frac{Y}{X}\right)=\frac{1}{1+e^{-\left(\alpha +\sum \beta_{i}X_{i}\right)}}$$

(5) $$\mathrm{where}\;z=\alpha +\beta_{i}X_{i}+\beta_{2}X_{2}+\ldots +\beta_{k}X_{k}$$

### 2.7. Evaluation Metrics

The classification performance of the machine learning classifiers was compared using five evaluation metrics. To evaluate the experimental results obtained from the model, the true positive (TP), true negative (TN), false negative (FN), and false positive (FP) values were calculated using the confusion matrix. The ratio of correctly classified samples was calculated based on TP and TN values. In addition, incorrectly classified samples were indicated by FN and FP. Finally, we obtained four indicators: precision, recall, f1-score, and accuracy. Furthermore, the true positive rate (TPR) and false positive rate (FPR) were determined to establish the receiver operating characteristic (ROC) curve. The area under the curve (AUC) was calculated from the ROC curve.

(6) $$\mathrm{Precision}=\frac{\mathrm{TP}}{\mathrm{TP}+\mathrm{FP}}$$

(7) $$\mathrm{Recall}=\frac{\mathrm{TP}}{\mathrm{TP}+\mathrm{FN}}$$

(8) $$F1-\mathrm{score}=2\times \frac{\mathrm{Precision}\times \mathrm{Recall}}{\mathrm{Precision}+\mathrm{Recall}}$$

(9) $$\mathrm{Accuracy}=\frac{\mathrm{TP}+\mathrm{TN}}{\mathrm{TP}+\mathrm{FP}+\mathrm{TN}+\mathrm{FN}}$$

(10) $$\mathrm{True}\;\mathrm{Positive}\;\mathrm{Rate}=\frac{\mathrm{TP}}{\mathrm{TP}+\mathrm{FN}}$$

(11) $$\mathrm{False}\;\mathrm{Positive}\;\mathrm{Rate}=\frac{\mathrm{FP}}{\mathrm{FP}+\mathrm{TN}}$$

### 2.8. Tools

All codes for ML classifiers and data preprocessing were written using Python (version 3.7.1; scikit-learn, version 2.4.1) and R (version 4.0.3) programming languages.

## 3. Results

### 3.1. Classification Performance Results from ML Classifiers

To identify risk factors using various variables, three machine-learning classifiers were applied to the preprocessed datasets. The classification performance results were compared using five evaluation metrics (precision, recall, f1-score, accuracy, and AUC). Among the three classifiers (XGBoost, SVC, and LR) used in this study, the XGBoost classifier demonstrated the best classification performance. In the experimental results, the highest evaluation metrics for both dependent variables were obtained from the integrated datasets. In addition, for the yearly datasets, the XGBoost classifier showed better performance than the other two algorithms. Details of the classification performance results are listed in Table 5, Table 6 and Table 7.

### 3.2. Feature Importance for the Identification of Risk Factors

Based on the classification performance results, important features from the trained XGBoost classifier were determined. The models with the best classification performances were selected to determine which features of the trained algorithms were important and reflected the characteristics of the variables. Common and unique factors associated with suicidal ideation and suicide attempts were identified and compared.

For the case with the integrated dataset, important features and their risk-factor scores are listed in Table 8. There are seven common variables with a high rank (ranked 1 to 7). Socioeconomic variables (e.g., average monthly income, age, drinking age, and education level) and nonmental health-related variables were identified as common variables. Next, the differences in ranks between the variables were examined for middle and low ranks (from rank 8 to 20). Unlike suicidal ideation-related risk factors, mental health-related variables (e.g., prevalence of depression, anxiety/depression in quality of life, and depression for more than 2 weeks) were ranked higher as being a risk factor for suicidal attempt.

In the results on the yearly dataset conditions, trends similar to those of the integrated dataset conditions were identified. The detailed results of the yearly dataset conditions are listed in Table 9, Table 10 and Table 11 and Supplementary Tables S1–S5. In addition, the suicide rate was analyzed by year, together with the experimental results. First, socioeconomic variables, including average monthly income, were found among the high ranking common variables of the two dependent variables. Second, similar to the analysis results obtained from the previously integrated dataset, mental health-related variables were confirmed to be ranked relatively high for risk of suicidal attempt. Third, considering the suicide rate, it was verified that the aforementioned characteristics of mental health variables were more prominent. For example, in 2009, 2010, and 2011, when the suicide rate was relatively high (31.0% in 2009, 31.2% in 2010, and 31.7% in 2011), the prevalence of depression, subjective health status, and depression for more than 2 weeks were ranked higher than other results for the same socioeconomic variables in 13 years.

## 4. Discussion

In this study, the risk factors associated with suicidal ideation and attempts were compared using machine learning classifiers. To determine the important factors, machine-learning algorithms were utilized for dataset analysis. After confirming the related factors using the classification algorithm, a novel risk-factor score based on the rank and importance scores was calculated from the algorithm to evaluate the importance of variables. To investigate the differences in the importance of factors, based on suicide risk level, suicide ideation was set as having a low suicide risk and suicide attempt was set as having a high suicide risk, prior to analyzing the results. In the experimental results, we found that the associations of socioeconomic and sociodemographic variables were high for both suicide ideation and attempt. In addition, the risk-factor scores of mental health variables were higher than those for other variables for the high suicide risk condition (i.e., suicide attempt).

Reasonable evidence was gathered from previous studies on the research topics (i.e., suicidal risk factors, suicidal ideation, suicidal attempts, and machine learning algorithms) before conducting our study. First, with regard to suicidal ideation and risk factors, Hintikka et al. [38] analyzed a 3-year prospective follow-up dataset collected from people living in Finland (*n* = 1339) to identify factors associated with suicidal ideation. From a longitudinal follow-up dataset, the authors focused on the risk factors of suicidal ideation. The impacts of sociodemographic and socioeconomic factors, including lifestyle, were identified for suicidal ideation. Weber et al. [39] examined the relationship between suicidal ideation and the diverse variables in a population sampled from college students. Among the variables used in this study, depression- and hopelessness-related variables showed a strong association with the main dependent variable (suicidal ideation). Kleiman et al. [40] attempted to identify related risk factors and their degree of variation in suicidal ideation within a short period of time. Well-known risk factors for suicidal ideation, such as hopelessness, burdensomeness, and loneliness, were varied and correlated with suicidal ideation. Second, many previous studies have associated various risk factors with suicide attempts. For example, Beautrais et al. [41] applied case–control designed datasets collected from 129 young people who had made serious suicide attempts. Among the various factors, the contributions of the risk factors of childhood adversity, social disadvantage, and psychiatric morbidity were found to be significant in the analysis results. Teti et al. [42] systematically reviewed several published studies to find similar risk factors for suicide among Latin American and Caribbean people. Major depressive disorder, family dysfunction, and prior suicide attempts were confirmed as the main risk factors. Parra-Uribe et al. [43] focused on the risk factors of suicide re-attempts and completed suicides after previous attempts. The authors identified the influences of alcohol use, personality disorders, and younger age on suicide re-attempts.

Finally, regarding the investigation of risk factors using machine learning algorithms, there were several previous studies on the detection of associated factors and suicide. Taneja et al. [44] applied a random forest classifier to predict the risk of sepsis from clinical variables in electronic medical record (EMR) datasets. The prediction model proposed that the “PCT” and “IL-6” variables are important in predicting risk of sepsis. Walsh et al. [45] applied machine learning algorithms to predict the risk of suicide attempts. A dataset comprising 5167 people was analyzed using the random forest algorithm. Among the predictors used in random forest models, non-fatal prior experience of suicide attempts, hospital utilization history, and visit tallies are the most important predictors. Colin et al. [46] used a machine-learning model to predict suicide attempts in adolescents. Longitudinal datasets collected from 974 adolescents over 17 years were used to investigate the effects of associated factors. Random forest algorithms were used to analyze the datasets. Among the feature importance results of predictors, the top 20 predictors were compared to evaluate their importance. Body mass index (BMI), age, anilide medications, propionic acid derivative medication, and selective serotonin reuptake inhibitors were identified as the top five important factors for suicide attempt prediction. A history of episodic mood disorder and other medication-related variables ranked relatively low in the experimental results.

Based on previous studies, we found it reasonable to conduct research on the selected topics of our study (i.e., identification of major risk factors for suicidal ideation and attempts with machine learning models). The experimental design of previous studies was adopted in our study. Unlike previous researchers who used datasets on specific patient groups, De la Garza et al. [31] used longitudinal datasets from a National US survey to investigate the characteristics of the general population. They divided the datasets based on intervals (e.g., from 2001 to 2002 was wave 1 and from 2004 to 2006 was wave 2) to compare the effects of the factors between periods. From the collected datasets, risk factors for suicide attempts were detected using a machine learning algorithm (random forest model). In wave 1, various variables, such as disorder-related variables (e.g., alcohol use, drug use, and nicotine dependence) and mood disorders (panic and bipolar disorder), were collected through interviews with participants. Three years after wave 1, in wave 2, the main dependent variables (i.e., non-fatal suicide attempts) were collected. A balanced random forest classifier was used to identify factors associated with suicide attempts through processed features in wave 1 and binary suicide attempts in wave 2. To quantify the importance of each variable, Youden J statistics were calculated to set the cut-off points for the evaluation metric values. Six evaluation indices (sensitivity, specificity, positive predictive value, negative predictive value, alarms per 100 evaluations, and number needed for evaluation) were used to examine the performance of the classification models. Among the 2985 available input features, the top 20 most important variables were compared. The authors found that individuals who “felt that they wanted to die” and “thought about committing suicide” showed the highest importance scores in the experimental results. In addition, the effects of socioeconomic disadvantages were observed in the analysis results.

Su et al. [47] used structured electronic health records (EHRs) from the Connecticut Children’s Medical Center (CCMC) for the 2011–2016 duration to predict suicidal risk in children and adolescents. From the CCMC EHR database, approximately 641,708 visits for 129,485 patients were extracted. To compare the model classification performances, several datasets with different conditions for the period were analyzed. The main dependent variables (i.e., suicide attempts) were identified using the International Classification of Diseases, Ninth Revision (ICD-9) code. In addition, demographics, prescribed medications, and clinical variables were included in the extracted datasets. The prediction model was evaluated using four evaluation indices (receiver operator characteristic curve, sensitivity, specificity, and positive predictive value). The variables were grouped and the performances of the prediction models were evaluated to confirm the usefulness of the predictive models proposed. The frequency of specific predictors was measured to confirm the influence of the variables on prediction. Symptoms and signs involving emotional state, depressive episode, and gender showed the highest rank among the input variables. In addition, antidepressant medications, including sertraline and escitalopram, and urine culture test variables were found to be highly important variables.

Design processes from previous studies were incorporated in our study, as described above. First, partial datasets with related variables were extracted from the KNHANES dataset from 1998 to 2019. For the common variables, only 13 datasets collected from 2007 to 2019 were selected. Second, the distributions of variables in the extracted datasets were examined to remove extreme or missing data. After removing more than half of the variables with missing data, 48 variables remained, including demographics, socioeconomic status, and mental health categories. Third, to select an optimized machine learning algorithm, the performances of the three classification models in our study were compared. Finally, the feature importance of the classifiers in identifying risk factors was determined for the best performance.

To compare the risk factors between suicide ideation and attempts, the importance of each variable in the datasets was evaluated with respect to suicide ideation and attempts. In addition, the conditions of the integrated dataset and year dataset were compared. The experimental results were analyzed, with suicidal ideation being a relatively low-risk group and suicidal attempt being a high-risk group. First, the results were compared from the perspective of the integrated dataset, regardless of the time-series, such as the year datasets. Socioeconomic and sociodemographic variables (e.g., average monthly income, age, education status, and drinking age) were confirmed to have a high rank and were common factors in both suicide ideation and attempt. In Ferretti and Coluccia [48], the trends in socioeconomic factors associated with determinants of suicide in the general population of Europe were similar, unlike that in the group of patients with mental illnesses. Mortensen et al. [49] suggested that a high risk of suicide was associated with unemployment and other socioeconomic factors in the Danish population. Among relatively low ranking factors, the rank of mental health-related variables (e.g., prevalence of depression, anxiety, depression related quality of life, and depression for more than 2 weeks) in high suicidal risk conditions (i.e., suicidal attempt) was higher than that in low-risk conditions (i.e., suicidal ideation). Based on these results, in the integrated dataset conditions, sociodemographic and socioeconomic factors are important for both suicidal ideation and attempts. In addition, mental health variables including depression or anxiety are risk factors for suicide attempts.

Second, the risk factors were investigated using yearly datasets with suicidal rate data to compare the factors for high and low suicide rates. From 2007 to 2019, the suicide rates in 2009, 2010, and 2011 were higher than in other years. Similar to the integrated dataset conditions, sociodemographic and socioeconomic factors were found to rank high for both ideation and attempt over 13 years. In datasets with a relatively high suicide rate, trends of depression prevalence and depression for more than two weeks ranking high were clearly identified. From these trends in the results, we confirmed that the yearly dataset with a high suicide rate showed similar trends to the results of the integrated datasets.

In conclusion, in terms of major risk factors for suicide, the analysis results based on a longitudinal dataset collected from the general population and analyzed using a machine learning classification model indicated that socioeconomic and sociodemographic factors were associated with both suicidal ideation and suicide attempts. Similar trends were validated on yearly datasets with yearly suicide rates, resulting in a high rank for social variables and a relatively higher rank for mental health variables associated with high suicide ranks.

## 5. Conclusions

In social and public health, an investigation of suicide risk factors is critical to solving or decreasing the impact of suicide on the public. In this study, we applied machine learning algorithms to identify risk factors for suicidal ideation and attempts using longitudinal datasets collected from the general population living in Korea. To compare the differences between suicidal ideation and suicidal attempt factors, the KNHANES dataset was preprocessed for two dependent variables (‘BP6_10′: suicide ideation and ‘BP6_31′: suicide attempt). In addition, datasets collected over 13 years (from 2007 to 2019) were analyzed to determine the associated risk factors for both the integrated dataset and datasets by year in terms of the importance of factors under different dataset conditions. Furthermore, to confirm the optimized machine learning algorithms for our research topic, we compared the performances of three machine learning classifiers (XGBoost classifier, support vector classifier, and logistic regression). Among the three classifiers, XGBoost showed the best performance on five evaluation metric values. Based on these results, we evaluated the feature importance of XGBoost in identifying important risk factors of suicidal ideation and attempt. As a common factor for ideation and attempts, sociodemographic and socioeconomic factors ranked high for various variables. In addition, we found that mental health variables showed a relatively high rank in the suicidal attempt condition, which was considered a high risk for suicide. From these experimental results, it was concluded that sociodemographic and socioeconomic factors are critical for suicide in the general population. In the high-risk group with suicidal attempts, mental health factors could also influence their suicide risk.

The first strength of this study was the application of machine learning algorithms to investigate the associated risk factors for suicidal ideation and suicide attempts. Second, the risk of each factor was determined using a new quantitative score calculated from the rank and importance scores calculated using machine learning algorithms. Third, large-scale real-world datasets collected from people living in Korea were utilized to reflect practical tendencies. Finally, the influence of risk factors on suicide ideation and attempts were evaluated and compared through conditions for risk of suicide. Our study has some limitations. First, we extracted and utilized several variables without considering a wide range of factors. However, we tried to select variables that were associated with suicide in previous studies. Second, various methodologies, including deep learning algorithms for the analysis of factors, can be applied to address our research questions. In our study, ML algorithms were used to facilitate the confirmation of feature importance. Third, additional comparisons and external validation will be required in future studies required on datasets collected from other countries to generalize the results.

## Figures and Tables

**Table 1:** Categories of variables in the KNHANES dataset.

No	Categories	Type of Variables
1	Health behavior	Categorical
2	Blood pressure measurement	Continuous
3	Blood test	Continuous
4	Grip strength test	Continuous
5	Dietary life survey	Categorical
6	Food safety investigation	Categorical
7	Food intake frequency survey	Categorical
8	Food intake survey	Continuous
9	Dietary life evaluation index	Continuous

**Table 2:** Baseline characteristics of the KNHANES dataset.

Characteristic		KNHANES
Age (years), mean (SD)		48.5 (18.0)
No. of participants (*n*)		78,796
Gender, *n* (%)	Male	34,230 (43.5%)
	Female	44,566 (56.5%)
Height (cm), mean (SD)		156.1 (19.7)
Weight (kg), mean (SD)		57.1 (18.0)
BMI, mean (SD)		22.6 (4.2)

**Table 3:** Dimensions and number of participants for datasets from 2007 to 2019.

Year	Dimension (# of Columns, # of Rows)	No. of Participants
2007	(2839, 49)	2839
2008	(6585, 49)	6585
2009	(7399, 49)	7399
2010	(6175, 49)	6175
2011	(5977, 49)	5977
2012	(6125, 49)	6125
2013	(5941, 49)	5941
2014	(5655, 49)	5655
2015	(5899, 49)	5899
2016	(6542, 49)	6542
2017	(6608, 49)	6608
2018	(6403, 49)	6403
2019	(6648, 49)	6648

**Table 4:** Hyperparameters applied in the machine learning classifiers.

Algorithm	Hyperparameter	Value (Argument)
XGBoost classifier	Eta	0.3
	Gamma	0
	max_depth	6
	min_child_weight	1
Support vector classifier	Kernel	rbf
	Gamma	auto
Logistic regression	Penalty	L2
	Solver	newton-cg

**Table 5:** Classification performance results for classifiers with yearly datasets (2007–2013).

Year	Dependent Variable	Classifier	Precision	Recall	F1-Score	Accuracy	AUC ^1^
2007	BP6_10 ^2^	XGBoost	0.850	0.866	0.859	0.886	0.920
		SVC ^4^	0.846	0.605	0.600	0.856	0.843
		LR ^5^	0.707	0.711	0.727	0.816	0.859
	BP6_31 ^3^	XGBoost	0.883	0.935	0.911	0.935	0.958
		SVC	0.868	0.530	0.523	0.658	0.656
		LR	0.527	0.600	0.595	0.758	0.798
2008	BP6_10	XGBoost	0.869	0.841	0.851	0.887	0.893
		SVC	0.721	0.586	0.523	0.815	0.805
		LR	0.704	0.758	0.706	0.784	0.829
	BP6_31	XGBoost	0.893	0.941	0.911	0.938	0.955
		SVC	0.471	0.506	0.485	0.459	0.649
		LR	0.530	0.600	0.496	0.710	0.692
2009	BP6_10	XGBoost	0.894	0.857	0.879	0.883	0.898
		SVC	0.743	0.648	0.644	0.824	0.807
		LR	0.688	0.737	0.708	0.783	0.828
	BP6_31	XGBoost	0.908	0.933	0.913	0.932	0.958
		SVC	0.549	0.537	0.484	0.419	0.731
		LR	0.551	0.682	0.567	0.751	0.761
2010	BP6_10	XGBoost	0.916	0.924	0.911	0.937	0.920
		SVC	0.726	0.554	0.497	0.840	0.809
		LR	0.676	0.757	0.698	0.806	0.833
	BP6_31	XGBoost	0.906	0.937	0.913	0.937	0.963
		SVC	0.667	0.540	0.527	0.814	0.725
		LR	0.550	0.673	0.565	0.763	0.752
2011	BP6_10	XGBoost	0.948	0.965	0.948	0.959	0.925
		SVC	0.789	0.533	0.523	0.817	0.830
		LR	0.685	0.782	0.709	0.818	0.848
	BP6_31	XGBoost	0.913	0.945	0.925	0.942	0.956
		SVC	0.469	0.503	0.487	0.728	0.637
		LR	0.509	0.589	0.577	0.692	0.601
2012	BP6_10	XGBoost	0.943	0.954	0.937	0.943	0.924
		SVC	0.793	0.860	0.799	0.861	0.786
		LR	0.857	0.791	0.801	0.790	0.819
	BP6_31	XGBoost	0.919	0.951	0.932	0.951	0.965
		SVC	0.476	0.500	0.488	0.865	0.710
		LR	0.535	0.644	0.515	0.765	0.731
2013	BP6_10	XGBoost	0.930	0.948	0.936	0.948	0.978
		SVC	0.538	0.507	0.499	0.525	0.833
		LR	0.586	0.778	0.602	0.833	0.842
	BP6_31	XGBoost	0.985	0.992	0.988	0.992	0.988
		SVC	0.496	0.500	0.498	0.604	0.852
		LR	0.519	0.800	0.502	0.864	0.826

^1^ AUC: area under curve; ^2^ BP6_10: suicidal ideation in the previous year; ^3^ BP6_31: suicide attempts within the last year; ^4^ SVC: support vector classifier; ^5^ LR: logistic regression.

**Table 6:** Classification performance results for classifiers with yearly datasets (2014–2019).

Year	Dependent Variable	Classifier	Precision	Recall	F1-Score	Accuracy	AUC ^1^
2014	BP6_10 ^2^	XGBoost	0.914	0.953	0.932	0.953	0.981
		SVC ^4^	0.475	0.500	0.487	0.516	0.865
		LR ^5^	0.587	0.749	0.599	0.839	0.828
	BP6_31 ^3^	XGBoost	0.960	0.980	0.969	0.980	0.977
		SVC	0.490	0.500	0.495	0.785	0.687
		LR	0.560	0.677	0.585	0.683	0.745
2015	BP6_10	XGBoost	0.927	0.943	0.932	0.943	0.980
		SVC	0.653	0.555	0.549	0.464	0.851
		LR	0.616	0.817	0.646	0.858	0.870
	BP6_31	XGBoost	0.983	0.991	0.987	0.991	0.989
		SVC	0.496	0.500	0.498	0.535	0.742
		LR	0.518	0.757	0.501	0.844	0.854
2016	BP6_10	XGBoost	0.917	0.942	0.929	0.955	0.989
		SVC	0.481	0.500	0.490	0.637	0.797
		LR	0.593	0.743	0.600	0.746	0.888
	BP6_31	XGBoost	0.988	0.994	0.991	0.994	0.990
		SVC	0.497	0.500	0.498	0.884	0.789
		LR	0.509	0.676	0.479	0.850	0.744
2017	BP6_10	XGBoost	0.939	0.952	0.941	0.952	0.980
		SVC	0.501	0.501	0.491	0.652	0.866
		LR	0.605	0.811	0.634	0.861	0.881
	BP6_31	XGBoost	0.988	0.994	0.991	0.994	0.990
		SVC	0.497	0.500	0.498	0.395	0.862
		LR	0.513	0.730	0.494	0.862	0.844
2018	BP6_10	XGBoost	0.918	0.958	0.938	0.958	0.972
		SVC	0.479	0.500	0.498	0.583	0.837
		LR	0.560	0.725	0.564	0.838	0.829
	BP6_31	XGBoost	0.984	0.992	0.988	0.992	0.990
		SVC	0.497	0.500	0.498	0.836	0.876
		LR	0.512	0.712	0.485	0.852	0.864
2019	BP6_10	XGBoost	0.937	0.950	0.939	0.950	0.982
		SVC	0.475	0.500	0.487	0.504	0.866
		LR	0.599	0.805	0.624	0.851	0.879
	BP6_31	XGBoost	0.991	0.995	0.993	0.995	0.990
		SVC	0.498	0.500	0.499	0.549	0.806
		LR	0.509	0.727	0.490	0.872	0.821

^1^ AUC: area under curve; ^2^ BP6_10: suicidal ideation in the previous year; ^3^ BP6_31: suicide attempts within the last year; ^4^ SVC: support vector classifier; ^5^ LR: logistic regression.

**Table 7:** Classification performance results for classifiers with integrated datasets.

Dependent Variable	Classifier	Precision	Recall	F1-score	Accuracy	AUC ^1^
BP6_10 ^2^	XGBoost	0.874	0.893	0.878	0.893	0.950
	SVC ^4^	0.442	0.500	0.470	0.885	0.811
	LR ^5^	0.653	0.779	0.677	0.808	0.853
BP6_31 ^3^	XGBoost	0.977	0.986	0.981	0.986	0.990
	SVC	0.493	0.500	0.497	0.682	0.766
	LR	0.524	0.794	0.493	0.805	0.850

^1^ AUC: area under curve; ^2^ BP6_10: suicidal ideation in the previous year; ^3^ BP6_31: suicide attempts within the last year; ^4^ SVC: support vector classifier; ^5^ LR: logistic regression.

**Table 8:** Important features and risk-factor score in the integrated dataset.

Rank	DV	Variable	Variable Description	Risk Variable Score	DV	Variable	Variable Description	Risk-Factor Score
1	BP6_10 ^1^	ainc	Average monthly income	0.2023	BP6_31 ^2^	ainc	Average monthly income	0.2030
2		age	Age of participant	0.1653		age	Age of participant	0.1472
3		BD2	Drinking age	0.1088		BD2	Drinking age	0.1107
4		BP8	Average sleep time per day	0.0729		BP8	Average sleep time per day	0.0969
5		educ	Education level	0.0518		educ	Education level	0.0392
6		BO1	Subjective body type recognition	0.4209		BO1	Subjective body type recognition	0.0389
7		D_1_1	Subjective health status	0.0377		D_1_1	Subjective health status	0.0330
8		BP1	Awareness of usual stress	0.0301		BO1_1	Weight change in past 1 year	0.0292
9		BO1_1	Weight change in past 1 year	0.0277		BP1	Awareness of usual stress	0.0274
10		incm	Personal income	0.0244		house	Home ownership	0.0231
11		house	Home ownership	0.0244		DF2_lt	Prevalence of depression	0.0216
12		DF2_lt	Prevalence of depression	0.0220		LQ_5EQL	EuroQoL: anxiety/depression	0.0208
13		EC1_1	Economic activity	0.0202		incm	Personal income	0.0208
14		LQ_4EQL	EuroQoL: pain/discomfort	0.0188		LQ_4EQL	EuroQoL: pain/discomfort	0.0190
15		ho_incm	Household income	0.0178		BP5	Depression for 2 weeks or more	0.0189
16		D_2_1	Uncomfortable experience in past 2 weeks	0.0167		EC1_1	Economic activity	0.0171
17		sex	Sex of participant	0.0166		sex	Sex of participant	0.0163
18		LQ_5EQL	EuroQoL: anxiety/depression	0.0149		D_2_1	Uncomfortable experience in past 2 weeks	0.0140
19		BP5	Depression for 2 weeks or more	0.0142		ho_incm	Household income	0.0140
20		LQ_1EQL	EuroQoL: athletic ability	0.0111		LQ_3EQL	EuroQoL: daily activity	0.0118

^1^ BP6_10: suicide ideation within the last year; ^2^ BP6_31: suicide attempts within the last year.

**Table 9:** Important features and risk-factor score in yearly dataset condition (2009).

Rank	DV	Variable	Variable Description	Risk Variable Score	DV	Variable	Variable Description	Risk-Factor Score
1	BP6_10 ^1^	ainc	Average monthly income	0.1700	BP6_31 ^2^	age	Age of participant	0.2000
2		age	Age of participant	0.1651		ainc	Average monthly income	0.1545
3		LQ_VAS	EuroQoL: total score	0.1168		LQ_VAS	EuroQoL: total score	0.1241
4		BD2	Drinking age	0.1045		BD2	Drinking age	0.1049
5		BP8	Average sleep time per day	0.0516		BP8	Average sleep time per day	0.0337
6		educ	Education level	0.0447		educ	Education level	0.0299
7		BO1	Subjective body type recognition	0.0375		BO1_1	Weight change in past 1 year	0.0297
8		D_1_1	Subjective health status	0.0350		BO1	Subjective body type recognition	0.0297
9		BO1_1	Weight change in past 1 year	0.0310		DF2_lt	Prevalence of depression	0.0278
10		BP1	Awareness of usual stress	0.0288		incm	Personal income	0.0248
11		incm	Personal income	0.0266		ho_incm	Household income	0.0231
12		house	Home ownership	0.0228		LQ_5EQL	EuroQoL: anxiety/depression	0.0210
13		EC1_1	Economic activity	0.0203		house	Home ownership	0.0202
14		ho_incm	Household income	0.0184		BP5	Depression for 2 weeks or more	0.0168
15		sex	Sex of participant	0.0147		EC1_1	Economic activity	0.0161
16		LQ_4EQL	EuroQoL: pain/discomfort	0.0136		D_1_1	Subjective health status	0.0159
17		BP5	Depression for 2 weeks or more	0.0135		LQ4_22	Activity restriction: old age	0.0145
18		D_2_1	Uncomfortable experience in past 2 weeks	0.0127		D_2_1	Uncomfortable experience in past 2 weeks	0.0134
19		DF2_lt	Prevalence of depression	0.0114		LQ_4EQL	EuroQoL: pain/discomfort	0.0125
20		LQ_1EQL	EuroQoL: athletic ability	0.0085		LQ_1EQL	EuroQoL: athletic ability	0.0097

^1^ BP6_10: suicide ideation within the last year; ^2^ BP6_31: suicide attempts within the last year.

**Table 10:** Important features and risk-factor score in yearly dataset condition (2010).

Rank	DV	Variable	Variable Description	Risk Variable Score	DV	Variable	Variable Description	Risk-Factor Score
1	BP6_10 ^1^	ainc	Average monthly income	0.2007	BP6_31 ^2^	ainc	Average monthly income	0.2158
2		age	Age of participant	0.1594		age	Age of participant	0.1703
3		LQ_VAS	EuroQoL: total score	0.1232		LQ_VAS	EuroQoL: total score	0.1505
4		BD2	Drinking age	0.1014		BD2	Drinking age	0.1420
5		BP8	Average sleep time per day	0.0486		BP8	Average sleep time per day	0.0342
6		educ	Education level	0.0442		D_1_1	Subjective health status	0.0223
7		BO1	Subjective body type recognition	0.0372		BO1	Subjective body type recognition	0.0197
8		D_1_1	Subjective health status	0.0305		educ	Education level	0.0196
9		BP1	Awareness of usual stress	0.0258		LQ4_08	Activity restriction: high blood pressure	0.0186
10		BO1_1	Weight change in past 1 year	0.0248		BO1_1	Weight change in past 1 year	0.0185
11		house	Home ownership	0.0222		BP1	Awareness of usual stress	0.0184
12		incm	Personal income	0.0212		incm	Personal income	0.0163
13		EC1_1	Economic activity	0.0206		LQ_5EQL	EuroQoL: anxiety/depression	0.0156
14		LQ4_08	Activity restriction: high blood pressure	0.0175		DF2_lt	Prevalence of depression	0.0145
15		D_2_1	Uncomfortable experience in past 2 weeks	0.0156		LQ4_10	Activity restriction: cancer	0.0136
16		LQ_4EQL	EuroQoL: pain/discomfort	0.0156		sex	Sex of participant	0.0133
17		ho_incm	Household income	0.0143		house	Home ownership	0.0119
18		sex	Sex of participant	0.0140		BP5	Depression for 2 weeks or more	0.0119
19		BP5	Depression for 2 weeks or more	0.0140		EC1_1	Economic activity	0.0102
20		DF2_lt	Prevalence of depression	0.0114		LQ4_01	Activity restriction: fracture/joint injury	0.0098

^1^ BP6_10: suicide ideation within the last year; ^2^ BP6_31: suicide attempts within the last year.

**Table 11:** Important features and risk-factor score in the yearly dataset condition (2011).

Rank	DV	Variable	Variable Description	Risk Variable Score	DV	Variable	Variable Description	Risk-Factor Score
1	BP6_10 ^1^	ainc	Average monthly income	0.2107	BP6_31 ^2^	ainc	Average monthly income	0.2338
2		age	Age of participant	0.1574		age	Age of participant	0.2127
3		LQ_VAS	EuroQoL: total score	0.1208		LQ_VAS	EuroQoL: total score	0.1690
4		BD2	Drinking age	0.1085		BD2	Drinking age	0.0694
5		BP8	Average sleep time per day	0.0482		ho_incm	Household income	0.0412
6		educ	Education level	0.0400		educ	Education level	0.0281
7		BO1	Subjective body type recognition	0.0336		BP8	Average sleep time per day	0.0261
8		D_1_1	Subjective health status	0.0270		BO1	Subjective body type recognition	0.0203
9		BP1	Awareness of usual stress	0.0264		BO1_1	Weight change in past 1 year	0.0185
10		BO1_1	Weight change in past 1 year	0.0245		DF2_lt	Prevalence of depression	0.0169
11		incm	Personal income	0.0244		EC1_1	Economic activity	0.0163
12		EC1_1	Economic activity	0.0207		D_1_1	Subjective health status	0.0152
13		house	Home ownership	0.0168		BP1	Awareness of usual stress	0.0152
14		sex	Sex of participant	0.0168		LQ_5EQL	EuroQoL: anxiety/depression	0.0136
15		BP5	Depression for 2 weeks or more	0.0159		D_2_1	Uncomfortable experience in past 2 weeks	0.0117
16		ho_incm	Household income	0.0155		BP5	Depression for 2 weeks or more	0.0116
17		D_2_1	Uncomfortable experience in past 2 weeks	0.0143		incm	Personal income	0.0094
18		LQ_4EQL	EuroQoL: pain/discomfort	0.0143		LQ1_sb	Lying in a sickbed in past 1 month	0.0083
19		DF2_lt	Prevalence of depression	0.0111		LQ_4EQL	EuroQoL: pain/discomfort	0.0082
20		LQ_5EQL	EuroQoL: anxiety/depression	0.0094		LQ4_05	Activity restriction: breathing problem/lung disease	0.0077

^1^ BP6_10: suicide ideation within the last year; ^2^ BP6_31: suicide attempts within the last year.

## Data Availability

In this study, we used the open-source KNHANES dataset released by the Korea Disease Control and Prevention Agency (KDCA).

## Supplementary Materials

The following are available online at https://www.mdpi.com/article/10.3390/ijerph182312772/s1, Table S1: Important features and risk-factor score in yearly dataset condition (2007 and 2008), Table S2: Important features and risk-factor score in yearly dataset condition (2012 and 2013), Table S3: Important features and risk-factor score in yearly dataset condition (2014 and 2015), Table S4: Important features and risk-factor score in yearly dataset condition (2016 and 2017), Table S5: Important features and risk-factor scores in yearly dataset conditions (2018 and 2019).
