# Supplementary material for: Identification of Risk Factors for Suicidal Ideation and Attempt Based on Machine Learning Algorithms: A Longitudinal Survey in Korea (2007–2019)
Source: Int J Environ Res Public Health. 2021 Dec 3;18(23):12772. doi: 10.3390/ijerph182312772 (PMC8657265; doi:10.3390/ijerph182312772)
Supplement: Supplementary file 1 [file ijerph-18-12772-s001.zip › ijerph-1441808-SI.pdf]

Supplementary Materials

Table S1: Important features and risk variable score in yearly dataset condition (2007 and 2008).

Rank	DV	Variable	Variable description	Risk variable score	DV	Variable	Variable description	Risk variable score
1	BP6_10[1]	ainc	Average monthly income	0.2020	BP6_31[2]	age	Age of participant	0.2372
2		age	Age of participant	0.1869		ainc	Average monthly income	0.1562
3		BD2	Drinking age	0.1205		BD2	Drinking age	0.1337
4		LQ_VAS	EuroQoL: total score	0.0800		LQ4_21	Activity restriction: obesity	0.0677
5		BP8	Average sleep time per day	0.0611		LQ4_00	Whether activity is restricted	0.0491
6		educ	Education level	0.0529		educ	Education level	0.0387
7		BO1	Subjective body type recognition	0.0383		LQ_VAS	EuroQoL: total score	0.0329
8		D_1_1	Subjective health status	0.0316		incm	Personal income	0.0295
9		BP1	Awareness of usual stress	0.0285		LQ4_06	Activity restriction: stroke	0.0294
10		BO1_1	Weight change in past 1 year	0.0264		LQ4_13	Activity restriction: hearing problem	0.0222
11		incm	Personal income	0.0256		LQ_4EQL	EuroQoL: pain/discomfort	0.0207
12		house	Home ownership	0.0243		BO1_1	Weight change in past 1 year	0.0195
13		EC1_1	Economic activity	0.0234		ho_incm	Household income	0.0195
14		ho_incm	Household income	0.0203		LQ4_07	Activity restriction: diabetes	0.0193
15		LQ_4EQL	EuroQoL: pain/discomfort	0.0194		LQ4_09	Activity restriction: back and neck problem	0.0183
16		BP5	Depression for 2 weeks or more	0.0177		LQ_3EQL	EuroQoL: daily activity	0.0167
17		sex	Sex of participant	0.0163		LQ4_22	Activity restriction: old age	0.0165
18		LQ_5EQL	EuroQoL: anxiety/depression	0.0158		BP8	Average sleep time per day	0.0161
19		DF2_lt	Prevalence of depression	0.0139		LQ4_05	Activity restriction: breathing problem/lung disease	0.0150
20		D_2_1	Uncomfortable experience in past 2 weeks	0.0136		BO1	Subjective body type recognition	0.0146
1	BP6_10[1]	age	Age of participant	0.1738	BP6_31[2]	age	Age of participant	0.2402
2		ainc	Average monthly income	0.1574		ainc	Average monthly income	0.1561
3		BD2	Drinking age	0.1123		BD2	Drinking age	0.1190
4		LQ_VAS	EuroQoL: total score	0.0920		LQ_VAS	EuroQoL: total score	0.0564
5		BP8	Average sleep time per day	0.0565		BP8	Average sleep time per day	0.0372
6		educ	Education level	0.0462		BO1_1	Weight change in past 1 year	0.0348
7		BO1	Subjective body type recognition	0.0378		BO1	Subjective body type recognition	0.0330
8		D_1_1	Subjective health status	0.0373		incm	Personal income	0.0317
9		BO1_1	Weight change in past 1 year	0.0280		educ	Education level	0.0265
10		incm	Personal income	0.0278		sex	Sex of participant	0.0230
11		BP1	Awareness of usual stress	0.0272		EC1_1	Economic activity	0.0203
12		EC1_1	Economic activity	0.0231		LQ_5EQL	EuroQoL: anxiety/depression	0.0174

13	house	Home ownership	0.0226	D_1_1	Subjective health status	0.0172
14	ho_incm	Household income	0.0186	LQ_4EQL	EuroQoL: pain/discomfort	0.0171
15	LQ_4EQL	EuroQoL: pain/discomfort	0.0183	BP1	Awareness of usual stress	0.0166
16	D_2_1	Uncomfortable experience in past 2 weeks	0.0160	ho_incm	Household income	0.0153
17	sex	Sex of participant	0.0149	BP5	Depression for 2 weeks or more	0.0142
18	BP5	Depression for 2 weeks or more	0.0130	house	Home ownership	0.0125
19	LQ_5EQL	EuroQoL: anxiety/depression	0.0110	DF2_lt	Prevalence of depression	0.0114
20	DF2_lt	Prevalence of depression	0.0099	LQ4_10	Activity restriction: cancer	0.0111

[1]BP6_10: suicide ideation in the last year; [2]BP6_31: suicide attempts in the last year.

Table S2: Important features and risk variable score in yearly dataset condition (2012 and 2013).

Rank	DV	Variable	Variable description	Risk variable score	DV	Variable	Variable description	Risk variable score
1	BP6_10[1]	ainc	Average monthly income	0.2253	BP6_31[2]	age	Age of participant	0.2485
2		age	Age of participant	0.1511		ainc	Average monthly income	0.2374
3		LQ_VAS	EuroQoL: total score	0.1138		LQ_VAS	EuroQoL: total score	0.1522
4		BD2	Drinking age	0.0920		BD2	Drinking age	0.0809
5		BP8	Average sleep time per day	0.0508		BP8	Average sleep time per day	0.0333
6		educ	Education level	0.0385		educ	Education level	0.0277
7		BO1	Subjective body type recognition	0.0370		BP5	Depression for 2 weeks or more	0.0188
8		D_1_1	Subjective health status	0.0309		D_1_1	Subjective health status	0.0169
9		incm	Personal income	0.0251		D_2_1	Uncomfortable experience in past 2 weeks	0.0158
10		BO1_1	Weight change in past 1 year	0.0224		ho_incm	Household income	0.0155
11		BP1	Awareness of usual stress	0.0224		BO1	Subjective body type recognition	0.0138
12		sex	Sex of participant	0.0207		house	Home ownership	0.0130
13		house	Home ownership	0.0195		incm	Personal income	0.0130
14		EC1_1	Economic activity	0.0188		BP1	Awareness of usual stress	0.0128
15		ho_incm	Household income	0.0165		LQ_2EQL	EuroQoL: self care	0.0124
16		LQ_4EQL	EuroQoL: pain/discomfort	0.0160		BO1_1	Weight change in past 1 year	0.0106
17		BP5	Depression for 2 weeks or more	0.0136		DF2_lt	Prevalence of depression	0.0094
18		LQ_1EQL	EuroQoL: athletic ability	0.0125		LQ_4EQL	EuroQoL: pain/discomfort	0.0092
19		D_2_1	Uncomfortable experience in past 2 weeks	0.0121		LQ_1EQL	EuroQoL: athletic ability	0.0078
20		DF2_lt	Prevalence of depression	0.0107		LQ_5EQL	EuroQoL: anxiety/depression	0.0072
1	BP6_10[1]	ainc	Average monthly income	0.2397	BP6_31[2]	ainc	Average monthly income	0.3279
2		age	Age of participant	0.1854		age	Age of participant	0.1689

3		BD2	Drinking age	0.1069		BD2	Drinking age	0.1292
4		BP8	Average sleep time per day	0.0531		BP8	Average sleep time per day	0.0399
5		BO1	Subjective body type recognition	0.0384		BO1_1	Weight change in past 1 year	0.0340
6		educ	Education level	0.0367		D_1_1	Subjective health status	0.0279
7		BO1_1	Weight change in past 1 year	0.0354		LQ_5EQL	EuroQoL: anxiety/depression	0.0264
8		BP1	Awareness of usual stress	0.0303		BO1	Subjective body type recognition	0.0247
9		D_1_1	Subjective health status	0.0268		educ	Education level	0.0245
10		LQ_4EQL	EuroQoL: pain/discomfort	0.0265		incm	Personal income	0.0205
11		house	Home ownership	0.0261		BP5	Depression for 2 weeks or more	0.0204
12		LQ_5EQL	EuroQoL: anxiety/depression	0.0198		BP1	Awareness of usual stress	0.0192
13		EC1_1	Economic activity	0.0189		LQ_4EQL	EuroQoL: pain/discomfort	0.0187
14		incm	Personal income	0.0183		house	Home ownership	0.0181
15		BP5	Depression for 2 weeks or more	0.0177		sex	Sex of participant	0.0143
16		ho_incm	Household income	0.0155		BP5	Depression for 2 weeks or more	0.0116
17		D_2_1	Uncomfortable experience in past 2 weeks	0.0143		incm	Personal income	0.0094
18		LQ_3EQL	EuroQoL: daily activity	0.0143		LQ1_sb	Lying in a sickbed in past 1 month	0.0083
19		DF2_lt	Prevalence of depression	0.0111		LQ_4EQL	EuroQoL: pain/discomfort	0.0082
20		LQ_1EQL	EuroQoL: athletic ability	0.0094		LQ4_05	Activity restriction: breathing problem/lung disease	0.0077

[1]BP6_10: suicide ideation in the last year; [2]BP6_31: suicide attempts in the last year.

Table S3: Important features and risk variable score in yearly dataset condition (2014 and 2015).

Rank	DV	Variable	Variable description	Risk variable score	DV	Variable	Variable description	Risk variable score
1	BP6_10[1]	ainc	Average monthly income	0.5317	BP6_31[2]	ainc	Average monthly income	0.1669
2		D_1_1	Subjective health status	0.0788		BP8	Average sleep time per day	0.0744
3		ho_incm	Household income	0.0545		BD2	Drinking age	0.0115
4		BD2	Drinking age	0.0520		age	Age of participant	0.0110
5		age	Age of participant	0.0429		ho_incm	Household income	0.0095
6		BP8	Average sleep time per day	0.0421		D_1_1	Subjective health status	0.0082
7		BP1	Awareness of usual stress	0.0361		incm	Personal income	0.0078
8		BO1	Subjective body type recognition	0.0358		BO1	Subjective body type recognition	0.0073
9		BD1	Lifetime drinking experience	0.0285		BD1	Lifetime drinking experience	0.0068
10		sex	Sex of participant	0.0278		educ	Education level	0.0063
11		incm	Personal income	0.0198		BP5	Depression for 2 weeks or more	0.0057
12		BP5	Depression for 2 weeks or more	0.0108		house	Home ownership	0.0056

13		house	Home ownership	0.0105		sex	Sex of participant	0.0047
14		educ	Education level	0.0099		BP1	Awareness of usual stress	0.0040
15		D_2_1	Uncomfortable experience in past 2 weeks	0.0032		EC1_1	Economic activity	0.0022
16		allownc	Whether or not receiving basic livelihood	0.0015		BP7	Whether counseling for mental problem in past 1 year	0.0014
17		EC1_1	Economic activity	0.0011		LQ1_sb	Lying in a sickbed in past 1 month	0.0009
18		BP7	Whether counseling for mental problem in past 1 year	0.0009		D_2_1	Uncomfortable experience in past 2 weeks	0.0007
19		LQ1_sb	Lying in a sickbed in past 1 month	0.0004		allownc	Whether or not to receive basic livelihood	0.0003
20		LQ4_16	Activity restriction: mental retardation	0.0003		LQ4_00	Whether activity is restricted	0.0001
1	BP6_10[1]	ainc	Average monthly income	0.2373	BP6_31[2]	ainc	Average monthly income	0.2662
2		age	Age of participant	0.1696		age	Age of participant	0.2111
3		BD2	Drinking age	0.1249		BD2	Drinking age	0.1273
4		BP8	Average sleep time per day	0.0572		BP8	Average sleep time per day	0.0367
5		educ	Education level	0.0465		BO1_1	Weight change in past 1 year	0.0252
6		BO1	Subjective body type recognition	0.0392		D_1_1	Subjective health status	0.0247
7		BO1_1	Weight change in past 1 year	0.0309		educ	Education level	0.0244
8		BP1	Awareness of usual stress	0.0304		BO1	Subjective body type recognition	0.0217
9		D_1_1	Subjective health status	0.0271		LQ_2EQL	EuroQoL: self care	0.0210
10		EC1_1	Economic activity	0.0232		LQ_4EQL	EuroQoL: pain/discomfort	0.0208
11		incm	Personal income	0.0231		LQ_5EQL	EuroQoL: anxiety/depression	0.0207
12		sex	Sex of participant	0.0222		LQ_3EQL	EuroQoL: daily activity	0.0197
13		BP5	Depression for 2 weeks or more	0.0212		ho_incm	Household income	0.0185
14		LQ_4EQL	EuroQoL: pain/discomfort	0.0170		BP7	Whether counseling for mental problem in past 1 year	0.0180
15		house	Home ownership	0.0165		BP5	Depression for 2 weeks or more	0.0177
16		LQ_5EQL	EuroQoL: anxiety/depression	0.0160		BP1	Awareness of usual stress	0.0173
17		ho_incm	Household income	0.0111		EC1_1	Economic activity	0.0170
18		LQ_1EQL	EuroQoL: athletic ability	0.0097		LQ_1EQL	EuroQoL: athletic ability	0.0170
19		LQ_3EQL	EuroQoL: daily activity	0.0092		incm	Personal income	0.0169
20		D_2_1	Uncomfortable experience in past 2 weeks	0.0091		sex	Sex of participant	0.0146

[1]BP6_10: suicide ideation in the last year; [2]BP6_31: suicide attempts in the last year.

Table S4: Important features and risk variable score in yearly dataset condition (2016 and 2017).

**Rank**	**DV**	**Variable**	**Variable description**	**Risk variable score**	**DV**	**Variable**	**Variable description**	**Risk variable score**

1	BP6_10[1]	ainc	Average monthly income	0.6288	BP6_31[2]	ainc	Average monthly income	0.3126
2		BP8	Average sleep time per day	0.1163		age	Age of participant	0.2167
3		incm	Personal income	0.0457		BP8	Average sleep time per day	0.1178
4		age	Age of participant	0.0321		BD2	Drinking age	0.0985
5		BP5	Depression for 2 weeks or more	0.0312		educ	Education level	0.0272
6		BD2	Drinking age	0.0227		D_1_1	Subjective health status	0.0236
7		D_1_1	Subjective health status	0.0210		BO1_1	Weight change in past 1 year	0.0227
8		sex	Sex of participant	0.0173		BO1	Subjective body type recognition	0.0191
9		house	Home ownership	0.0142		BP1	Awareness of usual stress	0.0189
10		educ	Education level	0.0130		sex	Sex of participant	0.0156
11		EC1_1	Economic activity	0.0114		LQ_4EQL	EuroQoL: pain/discomfort	0.0143
12		BP1	Awareness of usual stress	0.0094		incm	Personal income	0.0112
13		ho_incm	Household income	0.0089		LQ_3EQL	EuroQoL: daily activity	0.0108
14		BO1	Subjective body type recognition	0.0059		LQ_2EQL	EuroQoL: self care	0.0088
15		BD1	Lifetime drinking experience	0.0048		BP7	Whether counseling for mental problem in past 1 year	0.0087
16		LQ1_sb	Lying in a sickbed in past 1 month	0.0033		LQ_5EQL	EuroQoL: anxiety/depression	0.0084
17		D_2_1	Uncomfortable experience in past 2 weeks	0.0026		EC1_1	Economic activity	0.0083
18		BP7	Whether counseling for mental problem in past 1 year	0.0021		LQ_1EQL	EuroQoL: athletic ability	0.0082
19		allownc	Whether or not receiving basic livelihood	0.0004		house	Home ownership	0.0080
20		LQ4_10	Activity restriction: cancer	0.0016		D_2_1	Uncomfortable experience in past 2 weeks	0.0072
1	BP6_10[1]	ainc	Average monthly income	0.2332	BP6_31[2]	ainc	Average monthly income	0.2966
2		age	Age of participant	0.1562		age	Age of participant	0.2044
3		BP8	Average sleep time per day	0.1164		BD2	Drinking age	0.1289
4		BD2	Drinking age	0.1117		BP8	Average sleep time per day	0.0880
5		educ	Education level	0.0451		BP1	Awareness of usual stress	0.0282
6		BO1	Subjective body type recognition	0.0355		LQ_5EQL	EuroQoL: anxiety/depression	0.0235
7		BO1_1	Weight change in past 1 year	0.0338		BP5	Depression for 2 weeks or more	0.0221
8		D_1_1	Subjective health status	0.0239		D_1_1	Subjective health status	0.0201
9		BP5	Depression for 2 weeks or more	0.0226		LQ_4EQL	EuroQoL: pain/discomfort	0.0176
10		BP1	Awareness of usual stress	0.0217		BO1_1	Weight change in past 1 year	0.0173
11		incm	Personal income	0.0209		educ	Education level	0.0167
12		LQ_4EQL	EuroQoL: pain/discomfort	0.0198		LQ_1EQL	EuroQoL: athletic ability	0.0167
13		house	Home ownership	0.0191		sex	Sex of participant	0.0149
14		EC1_1	Economic activity	0.0176		incm	Personal income	0.0141
15		LQ_5EQL	EuroQoL: anxiety/depression	0.0170		house	Home ownership	0.0131
16		sex	Sex of participant	0.0139		BP7	Whether counseling for mental problem in past 1 year	0.0123

17	D_2_1	Uncomfortable experience in past 2 weeks	0.0128		LQ4_16	Activity restriction: mental retardation	0.0106
18	ho_incm	Household income	0.0127		BO1	Subjective body type recognition	0.0105
19	LQ_1EQL	EuroQoL: athletic ability	0.0104		D_2_1	Uncomfortable experience in past 2 weeks	0.0077
20	LQ_3EQL	EuroQoL: daily activity	0.0069		ho_incm	Household income	0.0071

[1]BP6_10: suicide ideation in the last year; [2]BP6_31: suicide attempts in the last year.

Table S5: Important features and risk variable score in yearly dataset condition (2018 and 2019).

Rank	DV	Variable	Variable description	Risk variable score	DV	Variable	Variable description	Risk variable score
1	BP6_10[1]	ainc	Average monthly income	0.4320	BP6_31[2]	ainc	Average monthly income	0.3252
2		BP8	Average sleep time per day	0.2797		age	Age of participant	0.1669
3		ho_incm	Household income	0.0428		BP8	Average sleep time per day	0.1391
4		age	Age of participant	0.0361		BD2	Drinking age	0.1239
5		BD2	Drinking age	0.0337		educ	Education level	0.0357
6		BO1	Subjective body type recognition	0.0248		BO1_1	Weight change in past 1 year	0.0211
7		educ	Education level	0.0246		BO1	Subjective body type recognition	0.0192
8		BD1	Lifetime drinking experience	0.0175		ho_incm	Household income	0.0184
9		BP1	Awareness of usual stress	0.0161		EC1_1	Economic activity	0.0181
10		D_1_1	Subjective health status	0.0152		incm	Personal income	0.0180
11		incm	Personal income	0.0106		BP1	Awareness of usual stress	0.0159
12		sex	Sex of participant	0.0101		D_1_1	Subjective health status	0.0146
13		BP5	Depression for 2 weeks or more	0.0078		LQ_4EQL	EuroQoL: pain/discomfort	0.0131
14		house	Home ownership	0.0059		house	Home ownership	0.0127
15		BP7	Whether counseling for mental problem in past 1 year	0.0052		LQ_1EQL	EuroQoL: athletic ability	0.0073
16		D_2_1	Uncomfortable experience in past 2 weeks	0.0009		LQ_5EQL	EuroQoL: anxiety/depression	0.0071
17		LQ1_sb	Lying in a sickbed in past 1 month	0.0006		D_2_1	Uncomfortable experience in past 2 weeks	0.0071
18		EC1_1	Economic activity	0.0003		DF2_lt	Prevalence of depression	0.0057
19		allownc	Whether or not receiving basic livelihood	0.0002		LQ_3EQL	EuroQoL: daily activity	0.0048
20		LQ4_04	Activity restriction: heart disease	0.0015		BP7	Whether counseling for mental problem in past 1 year	0.0034
1	BP6_10[1]	ainc	Average monthly income	0.2568	BP6_31[2]	ainc	Average monthly income	0.2931
2		age	Age of participant	0.1697		age	Age of participant	0.1794
3		BD2	Drinking age	0.1091		BD2	Drinking age	0.1485
4		BP8	Average sleep time per day	0.0679		BP8	Average sleep time per day	0.0642

5	educ	Education level	0.0448	BO1	Subjective body type recognition	0.0254
6	BO1	Subjective body type recognition	0.0391	incm	Personal income	0.0250
7	BO1_1	Weight change in past 1 year	0.0288	BP5	Depression for 2 weeks or more	0.0230
8	D_1_1	Subjective health status	0.0254	LQ_1EQL	EuroQoL: athletic ability	0.0229
9	house	Home ownership	0.0253	educ	Education level	0.0228
10	BP1	Awareness of usual stress	0.0250	LQ_2EQL	EuroQoL: self care	0.0221
11	incm	Personal income	0.0232	BO1_1	Weight change in past 1 year	0.0186
12	EC1_1	Economic activity	0.0214	BD1	Lifetime drinking experience	0.0180
13	LQ_4EQL	EuroQoL: pain/discomfort	0.0208	D_1_1	Subjective health status	0.0148
14	BP5	Depression for 2 weeks or more	0.0206	LQ_5EQL	EuroQoL: anxiety/depression	0.0135
15	LQ_2EQL	EuroQoL: self care	0.0199	house	Home ownership	0.0131
16	sex	Sex of participant	0.0192	LQ_3EQL	EuroQoL: daily activity	0.0114
17	LQ_3EQL	EuroQoL: daily activity	0.0141	D_2_1	Uncomfortable experience in past 2 weeks	0.0106
18	LQ_1EQL	EuroQoL: athletic ability	0.0135	BP1	Awareness of usual stress	0.0101
19	LQ_5EQL	EuroQoL: anxiety/depression	0.0128	LQ_4EQL	EuroQoL: pain/discomfort	0.0091
20	ho_incm	Household income	0.0120	sex	Sex of participant	0.0089

[1]BP6_10: suicide ideation in the last year; [2]BP6_31: suicide attempts in the last year
